# Four Novel *PAX9* Variants and the *PAX9*-Related Non-Syndromic Tooth Agenesis Patterns

**DOI:** 10.3390/ijms23158142

**Published:** 2022-07-24

**Authors:** Haochen Liu, Hangbo Liu, Lanxin Su, Jinglei Zheng, Hailan Feng, Yang Liu, Miao Yu, Dong Han

**Affiliations:** Department of Prosthodontics, Peking University School and Hospital of Stomatology & National Center of Stomatology & National Clinical Research Center for Oral Diseases & National Engineering Research Center of Oral Biomaterials and Digital Medical Devices, Beijing 100081, China; lhc@bjmu.edu.cn (H.L.); liuhb7@126.com (H.L.); slancy1219@pku.edu.cn (L.S.); zjl_pkuhsc@163.com (J.Z.); kqfenghl@bjum.edu.cn (H.F.); pkussliuyang@bjmu.edu.cn (Y.L.)

**Keywords:** tooth agenesis, *PAX9* variants, functional studies, phenotypic analysis

## Abstract

The purpose of this research was to investigate and identify *PAX9* gene variants in four Chinese families with non-syndromic tooth agenesis. We identified pathogenic gene variants by whole-exome sequencing (WES) and Sanger sequencing and then studied the effects of these variants on function by bioinformatics analysis and in vitro experiments. Four novel *PAX9* heterozygous variants were identified: two missense variants (c.191G > T (p.G64V) and c.350T > G (p.V117G)) and two frameshift variants (c.352delC (p.S119Pfs*2) and c.648_649insC(p.Y217Lfs*100)). The bioinformatics analysis showed that these variants might be pathogenic. The tertiary structure analysis showed that these four variants could cause structural damage to PAX9 proteins. In vitro functional studies demonstrated that (1) the p.Y217Lfs*100 variant greatly affects mRNA stability, thereby affecting endogenous expression; (2) the p. S119Pfs* 2 variant impairs the subcellular localization of the nuclear expression of the wild-type PAX9 protein; and (3) the four variants (p.G64V, p.V117G, p.S119Pfs*2, and p.Y217Lfs*100) all significantly affect the downstream transcriptional activity of the *BMP4* gene. In addition, we summarized and analyzed tooth missing positions caused by *PAX9* variants and found that the maxillary second molar (84.11%) and mandibular second molar (84.11%) were the most affected tooth positions by summarizing and analyzing the *PAX9*-related non-syndromic tooth agenesis positions. Our results broaden the variant spectrum of the *PAX9* gene related to non-syndromic tooth agenesis and provide useful information for future genetic counseling.

## 1. Introduction

Tooth agenesis, also known as congenitally missing teeth, is one of the most common congenital abnormalities in man, and it can cause masticatory dysfunction, speech changes, aesthetic problems, and malocclusion [1]. Based on the number of missing teeth, tooth agenesis is classified as hypodontia (lack of one to five permanent teeth, excluding the third molars), oligodontia (lack of six or more permanent teeth, excluding the third molars), or anodontia (complete lack of teeth) [2]. Tooth agenesis can occur as a non-syndromic form or can occur as part of a genetic syndrome (syndromic form) [3]. The overall incidence of tooth agenesis (excluding third molars) has been reported to be approximately 2–10% in different geographic regions and ethnicities [4,5,6]. Many factors can lead to congenital tooth deficiency, among which genetic defects are a major factor [1].

To date, a number of genes have been found to be associated with non-syndromic tooth agenesis, including axin inhibition protein 2 (*AXIN2*), muscle segment homeobox 1 (*MSX1*), paired box 9 (*PAX9*), ectodysplasin A (*EDA*), wingless-type mouse mammary tumor virus integration site family member 10A (*WNT10A*), wingless-type mouse mammary tumor virus integration site family member 10B, low-density lipoprotein receptor-related protein 6 (*LRP6*), and opsin 3 (*OPN3*) [2,7,8,9,10,11,12,13,14,15,16]. As one of the earliest discovered pathogenic genes of tooth agenesis, *PAX9* has been a research hotspot [2].

The *PAX9* gene is located on chromosome 14 at cytogenetic location 14q13.3 and encodes a member of the paired box transcription factor, PAX9 [17]. PAX9, which contains an octapeptide, a pair of box domains, and a 128-amino acid-long paired-type homeodomain, plays a critical role in odontogenesis [18]. *Pax9*-deficient mice lack pharyngeal pouch derivatives and teeth and exhibit craniofacial and limb abnormalities [19]. *Pax9* is required for the mesenchymal expression of Bmp4, Msx1, and Lef1 [19]. So far, approximately 50 pathogenic variants have been detected, most of them located in the paired domain (PD) [16].

The goal of this study was to broaden the *PAX9* variant spectrum associated with non-syndromic tooth agenesis, investigate the functional implications of newly discovered novel variant loci, and analyze the genotype–phenotype relationships.

## 2. Materials and Methods

### 2.1. Subjects

Four probands with non-syndromic tooth agenesis were identified in the Department of Prosthodontics, Peking University School of Stomatology (Beijing, China). All the probands and available family members of four pedigrees (11 individuals) participated in this study. The exact position of missing teeth was revealed by oral examinations and panoramic dental radiographs. Missing teeth due to trauma or extraction were excluded. Other developmental abnormalities were confirmed via physical examinations. This study obtained informed consent from all participants and was approved by the School of Stomatology and Hospital Ethics Committee of Peking University (PKUSSIRB-202162021).

### 2.2. Variant Detection and Analysis

The genomic DNA of the participants was extracted from peripheral blood using a BioTek DNA Whole-blood Mini Kit (BioTek, Beijing, China) according to the manufacturer’s instructions. Whole-exome sequencing was performed to identify potential pathogenic variants by Beijing Angen Gene Medicine Technology (Beijing, China) with the Illumina-X10 platform by iGeneTech. To filter the detected variants, we annotated the orodental-related genes [20]. Then, we excluded silent variants and missense variants with a minor allele frequency (MAF) ≥ 0.01 in East Asians in the Genome Aggregation Database (gnomAD, http://gnomad.broadinstitute.org/ (accessed on 29 November 2021)), the single Nucleotide Polymorphism database (dbSNP, http://www.ncbi.nlm.nih.gov/projects/SNP/snpsummary.cgi/ (accessed on 29 November 2021)), the 1000 Genomes Project database (1000G, http://www.1000genomes.org (accessed on 29 November 2021)), or the Exome Aggregation Consortium (ExAC, http://exac.broadinstitute.org (accessed on 29 November 2021)).

For the bioinformatics analysis, Mutation Taster (https://www.mutationtaster.org/ (accessed on 7 January 2022)) was used to evaluate the disease-causing potential of these variants, and the functional effects and pathogenicity of two missense mutations were predicted using the Sorting Intolerant From Tolerant (SIFT; http://provean.jcvi.org/index.php (accessed on 7 January 2022)), Protein Variation Effect Analyzer (PROVEAN; http://provean.jcvi.org/index.php (accessed on 7 January 2022)), Polymorphism Phenotyping v2 (PolyPhen-2; http://genetics.bwh.harvard.edu/pph2/ (accessed on 7 January 2022)), and Functional Analysis through Hidden Markov Models (fathmm; http://fathmm.biocompute.org.uk/inherited.html (accessed on 7 January 2022)). 

For conservation analysis, the amino acid sequences of PAX9 among 10 different species were obtained from the UniprotKB database (https://www.ncbi.nlm.nih.gov/ (accessed on 7 January 2022)). MEGA 11.0 was used to conduct the multiple sequence alignment and sequence logos were performed with WebLogo V2.8.2 (http://weblogo.berkeley.edu/ (accessed on 7 January 2022)).

For tertiary structural analysis, the PAX9 protein structure was obtained from AlphaFold Protein Structure Database (https://alphafold.ebi.ac.uk/ (accessed on 7 January 2022)). PyMol v2.1 (Molecular Graphics System, DeLano Scientific, CA, USA) was used to visualize the three-dimensional (3D) structure to analyze the structural changes.

### 2.3. Sanger Sequencing and Clone Sequencing

We confirmed 4 novel pathogenic variants of *PAX9* (NM_001372076.1) and excluded other candidate genes in 4 affected families. Co-segregation analysis and Sanger sequencing of the probands and their family members were performed to verify the variants of *PAX9*. The exons and exon–intron boundaries of the *PAX9* gene of four families was amplified using polymerase chain reactions (PCR). The primer information and conditions for PCR are shown in Supplementary Table S1. The PCR products were sequenced by Tsingke Biological Technology (Beijing, China). TA clone sequencing was used to confirm the exact status of the frameshift variant.

### 2.4. Construction of Plasmids

The full-length coding sequence of wild-type *PAX9* (NM_001372076.1) was subcloned into the pEGFP-N1 expression vector with enhanced GFP to synthesize the wild-type plasmid pEGFP-PAX9. Site-directed mutagenesis was performed to generate four variant plasmids: pEGFP-PAX9-G64V, pEGFP-PAX9-V114G, pEGFP-PAX9-S119Pfs*2, and pEGFP-PAX9-Y217Lfs*100. The Beijing Genomic Institute (BGI, Beijing, China) synthesized all plasmids and confirmed the entire sequence of the variant constructs. The construction of p.2.4BMP4-Luc, the downstream luciferase reporter plasmid, was described in our previous work [16].

### 2.5. Cell Culture and Transfection

In the presence of 5% CO2, 293T cells were grown in Dulbecco’s modified Eagle’s medium (Invitrogen, Grand Island, NY, USA) with supplements of 10% fetal bovine serum and 2 mmol/L L-glutamine. Following the manufacturer’s instructions, transient transfection was carried out using Lipofectamine 3000 (Invitrogen).

### 2.6. Western Blot Analysis

Proteins from each group were harvested 48 h after transfection. Cell lysates containing 20 μg of total protein were used in the Western blot analysis. After electrophoresis on 10% polyacrylamide gel, the protein was transferred to a PVDF membrane by electrophoresis and then incubated with anti-GFP (ab1218) and anti-β-actin (ab8226) mouse antibodies (Abcam, Cambridge, UK). The membrane was washed and incubated with peroxidase-conjugate rabbit anti-mouse secondary antibodies (ab6728; Abcam).

### 2.7. Real-Time PCR and mRNA Stability Studies

Total RNA was isolated using a RNeasy plus mini kit (Qiagen, Germantown, MD, USA) 12 h after transfection. Then, a Qiagen reverse transcription kit was used for reverse transcription. A pair of specific primers, PAX9-F: 5′-AACCAGCTGGGAGGAGTGTT-3′ and PAX9-R: 5′-TGATGTCACGGTCGGATG-3′, were designed. They are located at the N-terminal of the paired box domain and can identify wild type and variant mRNA. The expression of PAX9 was normalized by eukaryotic 18S ribosomal RNA (rRNA). Cells were treated with 10 μg/mL actinomycin D (Gibco) 12 h after transfection. After 4 and 8 h of treatment, total RNA was harvested for reverse transcription. The stability of mRNA is expressed as the percentage of remaining *PAX9* mRNA, as measured by 18SrRNA.

### 2.8. Subcellular Localization Assay

We transiently transfected 293T cells with pEGFP-N1 expression plasmids containing GFP-tagged wild-type or variant *PAX9* cDNAs, and 48 h after transfection, the cells were washed three times with phosphate buffer and fixed with 4% paraformaldehyde for 15 min. The cells were washed three times with phosphate buffer and placed in a mounting medium with 4′,6-diamino-2-phenylindole (Solarbio, Beijing, China). An LSM 510 Meta confocal microscope (Zeiss, Oberkochen, Germany) with a ×40/1.00 numerical aperture oil objective lens was used to photograph.

### 2.9. Luciferase Reporter Assay

Each *PAX9* expression plasmid and empty vector was co-transfected with p.2.4 BMP4-Luc and a phRL-TK-Renilla luciferase vector. Cell lysates were collected 48 h after transfection. Firefly and Renilla luciferase activities were assayed through a dual-luciferase reporter assay system (Promega) according to the manufacturer’s instructions. The independent luciferase report experiment was conducted in triplicate. Firefly luciferase activity was normalized according to Renilla luciferase activity.

### 2.10. Statistical Analysis

Statistical analyses were performed with GraphPad Prism (V8.0). Student’s *t* test was used for mRNA expression, stability studies, and luciferase report tests. Quantitative results are expressed as mean ± standard deviation (SD), with *p* < 0.05 being considered statistically significant.

To analyze the non-syndromic tooth agenesis pattern in patients with *PAX9* variants, 5 patients with detailed missing tooth position records from this study and previously published studies were included. All the human *PAX9* variations involved were reported in the Human Gene Mutation Database (HGMD) and PubMed database. A total of 146 individuals from 37 studies had no systemic disease or other ectodermal abnormalities, and detailed missing tooth positions were recorded (Supplementary Table S2). The number of missing teeth in 151 patients from this study and previous reports was compiled at each position in the four quadrants. The percentage of missing teeth was counted to measure the congenital deficiency rate, and the rate of missing teeth at different positions in the same arch was analyzed by Chi-square test, with *p* < 0.05 considered statistically significant.

## 3. Results

### 3.1. Clinical Findings and Variant Detection

**Figure 1:**
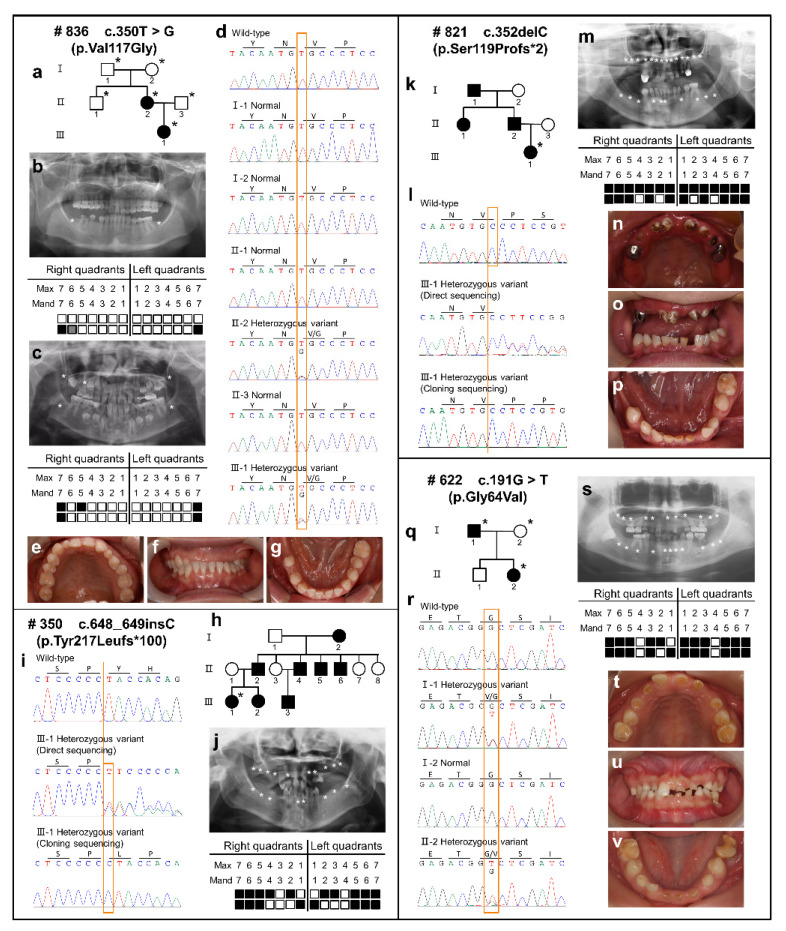
Pedigree, dental characteristics, and sequencing chromatograms of four families. (**a**) Pedigree of family #836. (**b**) Panoramic radiographs and schematic of #836 proband (II:1) with tooth agenesis. (**c**) Panoramic radiographs and schematic of #836 proband’s daughter (III:1) with tooth agenesis. (**d**) Sequencing chromatograms of available DNA in family #836 present a heterozygous *PAX9* missense variant (c.350T > G; p.Val117Gly) identified in the proband (II:1) and her father (III:1). (**e**–**g**) Digital photographs of #836 proband (II:1). (**h**) Pedigree of family #350. (**i**) Sequencing chromatograms of available DNA in family #350 present a heterozygous *PAX9* frameshift variant (c.648_649insC; p.Tyr217Leufs*100) identified in the proband (III:1). (**j**) Panoramic radiographs and schematic of #350 proband (III:1) with tooth agenesis. (**k**) Pedigree of family #821. (**l**) Sequencing chromatograms of available DNA in family #821 present a heterozygous PAX9 frameshift variant (c.352delC; p.Ser119Profs*2) identified in the proband (III:1). (**m**) Panoramic radiographs and schematic of #821 proband (III:1) with tooth agenesis. (**n**–**p**) Digital photographs of #821 proband (III:1). (**q**) Pedigree of family #622. (**r**) Sequencing chromatograms of available DNA in family #622 present a heterozygous *PAX9* missense variant (c.191G > T; p.Gly64Val) identified in the proband (II:2) and her father (I:1). (**s**) Panoramic radiographs and schematic of #622 proband (II:2) with tooth agenesis. (**t**–**v**) Digital photographs of #622 proband (II:2). The arrow in the pedigree indicates the proband. The asterisks in the pedigree represent participating family members. Squares and circles with a slash in the pedigree represent individuals who have passed away. Asterisks in panoramic radiographs and black squares in the schematics indicate congenital missing permanent teeth. Gray squares in the schematics indicate extracted teeth. Mand, mandibular; Max, maxillary.

The pedigrees, dental characteristics, and tooth agenesis patterns (excluding the third molars) of the probands are shown in Figure 1. Four novel heterozygous variants of the *PAX9* gene were identified by WES and confirmed by Sanger sequencing (Figure 1d,i,l,r). The variants were not identified in the 1000 Genomes, Exome Aggregation Consortium (ExAC), or gnomAD databases.

In family #836, the proband (II:2) was a 32-year-old female. Clinical and radiographical examinations revealed two missing mandibular second molars, and the shape of both maxillary lateral incisors were conic (Figure 1b,e–g). The loss of the right mandibular first molar was due to an extraction. Her daughter (III:1) was missing all second molars and the right maxillary second premolar (Figure 1c). Her parents (I:1 and I:2) and brother (II:1) were normal. The proband and her daughter carried a heterozygous missense variant (c.350T > G; p.Val117Gly/p.V117G) (Figure 1d). The variant was not identified in the proband’s parents and brother, indicating de novo.

In family #350, the proband (III:1) was a 26-year-old female. She was diagnosed with oligodontia due to the agenesis of 18 permanent teeth, including all molars, five premolars, one canine, and four incisors (Figure 1j). She provided a family history that showed that her grandmother (I:2), father (II:2), elder sister (III:2), three uncles (II:4, II:5, II:6), and one cousin (III:3) had the same phenotypes. A heterozygous frameshift variant (c.648_649insC; p.Tyr217Leufs*100/p.Y217Lfs*100) was identified in the proband (Figure 1i). Unfortunately, other members of the family were not available for genetic testing.

In family #821, the proband was a 26-year-old female. She had congenital absence of most permanent teeth except for her mandibular lateral incisors and first premolars (Figure 1m–p). The proband mentioned that her grandfather (I:1), father (II:2), and aunt (II:1) were congenitally missing permanent teeth. A heterozygous frameshift variant (c.352delC; p.Ser119Profs * 2/p.S119Pfs * 2) was identified in the proband (Figure 1l). Unfortunately, other members of the family were not available for genetic testing.

In family #622, the proband (II:2) was a 19-year-old female and had 22 missing permanent teeth. All first premolars and two incisors existed and most primary teeth of the proband were retained (Figure 1s–v). Her father (I:1) had the phenotype of non-syndromic tooth agenesis. The proband and her farther carried a heterozygous missense variant (c.191G > T; p.Gly64Val/p.G64V) (Figure 1r). According to family co-segregation, this variant was inherited by autosomal dominant inheritance.

### 3.2. Conservation and Bioinformatics Analysis

**Figure 2:**
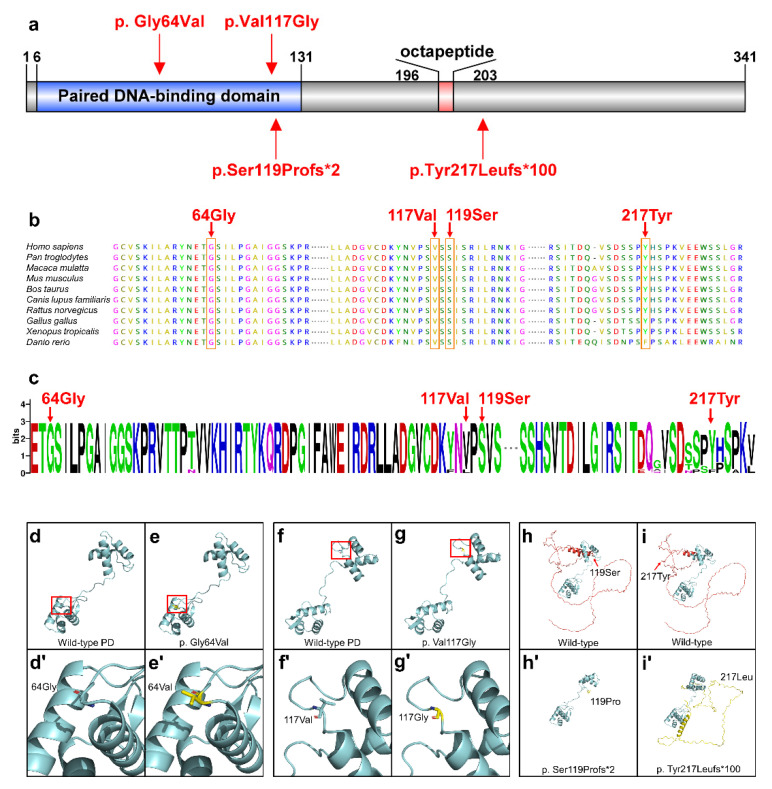
Conservation and bioinformatics analysis of the PAX9 variants. (**a**) Schematic diagram of the wild-type PAX9 protein and the localization of the 4 novel PAX9 variants identified in this study. (**b**) Conservation analysis of the PAX9 amino acid sequences among different species. (**c**) Weblogo analysis of the PAX9 amino acid sequences among different species. (**d**) 64Gly in wild-type PD. (**d’**) Higher magnifications of the above boxed regions surrounding the 64Gly. (**e**) Structural changes in p.Gly64Val in PD. (**e’**) Higher magnifications of the above boxed regions surrounding the 64Val. (**f**) 117Val in wild-type PD. (**f**’) Higher magnifications of the above boxed regions surrounding the 117Val. (**g**) Structural changes in p.Val117Gly in PD. (**g’**) Higher magnifications of the above boxed regions surrounding the 117Gly. (**h**) Structure of wild-type PAX9 (the amino acids behind 119Ser are shown in red). (**h’**) Structure of p.Ser119Profs*2 in PAX9 (the changed amino acids are shown in yellow). (**i**) Structure of wild-type PAX9 (the amino acids behind 217Tyr are shown in red). (**i’**) Structure of p.Tyr217Leufs*100 in PAX9 (the changed amino acids are shown in yellow).

In order to predict the harm of four novel *PAX9* variants, we performed conservative and bioinformatics analysis. Three variants (p.Gly64Val, p.Val117Gly, and p.Ser119Profs *2) were located in the highly conserved PD of PAX9 (Figure 2a). Four variants were predicted to be damaging or deleterious by SIFT, PROVEAN, PolyPhen-2, fathmm, or Mutation Taster, and they were all pathogenic according to the ACMG Classification [21] (Table 1). Based on the result of conservation analyses in multiple species, 64Gly, 117Val, 119Ser, and 217Tyr were highly conserved (Figure 2b,c).

A spatial structural analysis revealed that the residue at sequence position 64 in this protein is a glycine that has no side chain, giving the protein increased flexibility at this location (Figure 2d,d’). The variant (p.Gly64Val) residue is a valine that has a hydrophobic aliphatic side chain (Figure 2e,e’). For the variant p. Val117Gly, 117 sites changed from valine with a side chain to glycine without a side chain, which increased the affinity of protein at this position (Figure 2f,f’,g,g’). The p. Ser119Profs*2 variant changed the conformation of PD (Figure 2h,h’). The three-dimensional structure of the p.Tyr217Leufs *100 variant protein changed significantly (Figure 2i,i’).

### 3.3. Functional Analyses of PAX9 Variants

**Figure 3:**
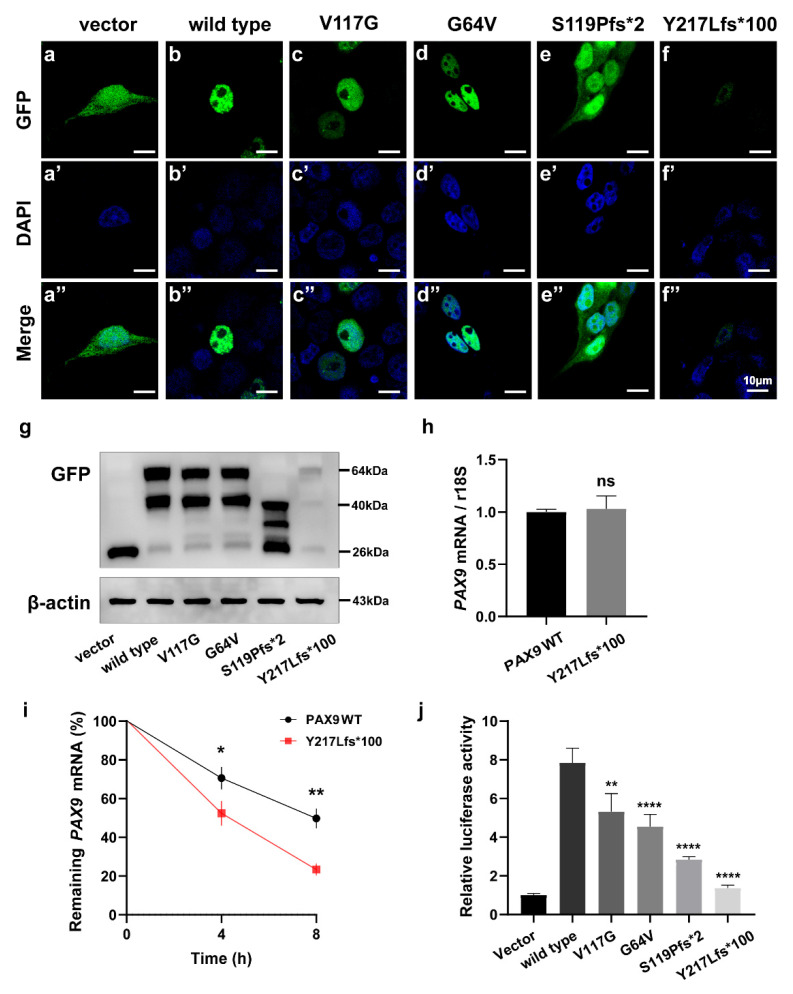
Functional studies of variant PAX9 proteins. (**a**–**f**) Subcellular localization of wild-type or variant PAX9 in vitro. (**a’**–**f’**) Nuclei staining by DAPI, 4,6-diamino-2-phenylindole. (**a″**–**f″**) Merging of GFP and DAPI. MSX1 (GFP, green); nuclei (DAPI, blue). (**g**) Western blot analysis of wild-type or variant PAX9 using anti-GFP and anti-β-actin antibodies. An empty vector was transfected as a negative control. (**h**) Relative messenger RNA (mRNA) expression levels of variant *PAX9* at 12 h after transfection. Data were statistically analyzed by the Student’s *t*-test. *n*=3. ns, no significance. (**i**) After treatment with actinomycin D, PAX9 mRNA underwent decay. The mRNA stability is presented by the percentage of remaining mRNAs at 4 and 8 h posttreatment. Data were statistically analyzed by the Student’s *t*-test. *n*=3. * *p* < 0.05. ** *p* < 0.01. (**j**) Transactivation activity of PAX9 variants on the BMP4 promoter by luciferase reporter assay. Data were statistically analyzed by the Student’s *t*-test. *n*=5. ** *p* < 0.01. **** *p* < 0.0001.

To confirm that these variants affect the function of PAX9, we performed in vitro studies using plasmids containing four novel PAX9 variants. Western blot (Figure 3g) showed that two variant proteins (p.G64V and p.V117G) caused by a missense variant and one truncated protein (p.S119Pfs*2) caused by a frameshift variant were expressed in vitro. However, one variant protein (p.Y217Lfs*100) caused by a frameshift variant (c.648_649insC) was almost undetectable. We then examined the mRNA expression level of *PAX9* variants. Baseline expression levels of truncated mRNA(p.Y217Lfs*100) did not differ from the wild-type *PAX9* (*p* > 0.05) (Figure 3h). After actinomycin D treatment, the percentage of residual mutant mRNA (p.Y217Lfs*100) was significantly lower than that of wild-type mRNA at 4 h (*p* < 0.05) and in 8 h (*p* < 0.01) (Figure 3i). This result indicates that this frameshift variant (c.648_649insC) expresses an unstable mRNA that is more susceptible to degradation compared to wild-type PAX9.

Subcellular localization showed that two missense variants (p.G64V and p.V117G) were located in the nucleus as well as in the wild type (Figure 3c–c″,d–d″), while the frameshift variant (p. S119Pfs*2) was located in the whole cytoplasm (Figure 3e–e″). The frameshift variant (p.Y217Lfs*100) was weakly expressed in the nucleus (Figure 3f–f″). The luciferase results showed that the transactivation capacity of PAX9 to the BMP4 promoter (one of the downstream targets of PAX9 during the process of tooth development) was significantly reduced in p.G64V, p.V117G, p.S119Pfs*2, and p.Y217Lfs*100 when compared with the wild-type group (*p* < 0.05) (Figure 3e).

### 3.4. Statistical Analysis of the PAX9-Related Non-Syndromic Tooth Agenesis Pattern

**Figure 4:**
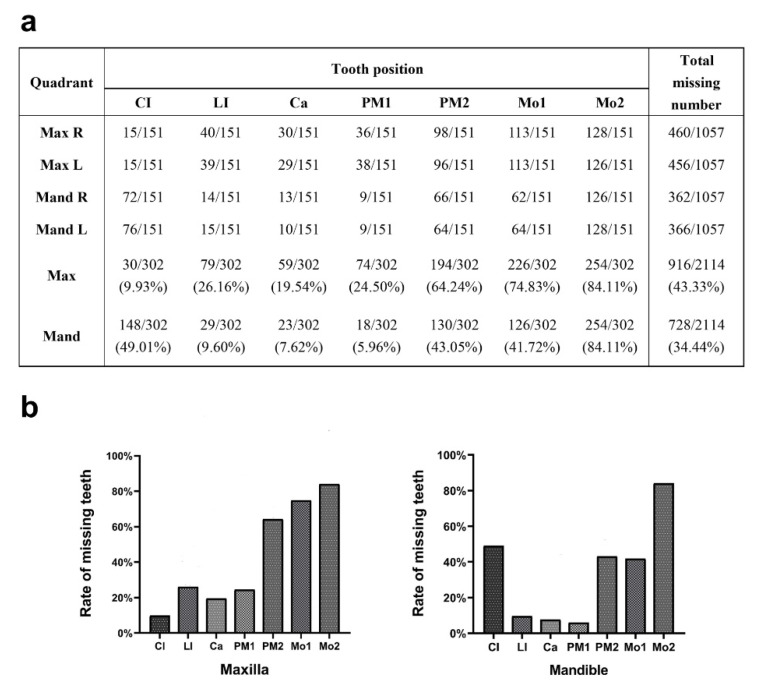
The pattern of PAX9-related non-syndromic tooth agenesis. (**a**) The number of missing teeth in 151 patients with *PAX9* variants. (**b**) The rate of missing teeth in 151 patients with *PAX9* variants was compiled at seven tooth positions in the maxillary and mandibular regions. The rate of missing teeth at different positions in the maxillary arch was statistically different using Pearson’s chi-squared test, *X*^2^ (6, N = 2114) = 666.98, *p* < 0.001. The rate of missing teeth at different positions in the mandibular arch was statistically different using Pearson’s chi-squared test, *X*^2^ (6, N = 2114) = 662.58, *p* < 0.001. Max, maxillary; Mand, mandibular; CI, central incisor; LI, lateral incisor; Ca, canine; PM1, first premolar; PM2, second premolar; Mo1, first molar; Mo2, second molar.

We collected 151 non-syndromic patients and recorded their *PAX9* variants, variant domains, and detailed missing teeth sites (Supplementary Table S2). The third molars and extracted teeth were excluded. The number of missing teeth of every position in four quadrants was calculated (Figure 4a). The average number of missing teeth was 10.89 and the missing rate was 38.88% (excluding the third molars). The percentage of missing teeth in the maxillary arch (43.33%) was higher than in the mandibular arch (34.44%) (*p* < 0.0001). The lower second molars (84.11%), upper second molars (84.11%), and upper first molars (74.83%) were the most affected, and the lower first premolars (5.96%), canines (7.62%), and lateral incisors (9.60%) were the least affected. Statistically significant differences (*p* < 0.05) were found among different positions in the same arch (Figure 4b).

## 4. Discussion

Non-syndromic tooth agenesis caused by *PAX9* variants is inherited by autosomal dominant inheritance [2]. In this work, we identified four novel *PAX9* variants in Chinese families with non-syndromic tooth agenesis, including two missense variants (p.G64V and p.V117G) and two frameshift variants (p.S119Pfs*2 and p.Y217Lfs*100). Although we studied two families (#350 and #821) in which we only had access to the proband’s sequencing results, the clinical phenotype, gene sequencing results, and pedigree map of these four families are consistent with the autosomal dominant inheritance model.

There are now 66 *PAX9* variations linked to non-syndromic tooth agenesis, with the majority being missense (34/66) and frameshift (21/66) variants. We discovered that 82.3% (28/34) of missense variants and 76.2% (16/21) of frameshift variants are identified in the PD. (Supplementary Table S2). In this study, two missense variants (p.G64V and p.V117G) were located in the PD, which supports the hypothesis that missense variants are more likely to be detected at ‘hotspot’ amino acids that are highly conserved and represent regions of structural or functional importance [22]. One frameshift variant (p.S119Pfs*2) was also located in the PD. Our results further confirm that the PD is a hotspot region for *PAX9* variants [16].

PD is a highly conserved functional domain. PAX9 protein regulates downstream DNA through PD, thereby affecting tooth development [23,24]. Three variants (p.G64V, p.V117G, and p.S119Pfs*2) were located in the highly conserved PD (Figure 2a–c). Our tertiary structural analysis revealed that p.V117G and p.G64V change the PD structure, whereas p.S119Pfs*2 leads to extreme structural disorders of the PD. These results suggest that these three variants may affect the DNA-binding ability of PAX9. Although p.Y217Lfs *100 is not located in the PD, tertiary structure analysis shows that the structure of the whole protein has undergone tremendous changes. It possible that the p.Y217Lfs*100 variant changed the function of PAX9.

The results of in vitro experiments further proved that these four variants may affect protein function. Western blot testing (Figure 3g) demonstrated that three variant proteins (p.G64V, p.V117G, and p.S119Pfs*2) were expressed in vitro; however, the p.Y217Lfs*100 variant protein was almost undetectable. The subsequent mRNA stability experiments proved that the p.Y217Lfs*100 variant greatly affected mRNA stability. In the last few years, it has become evident that mRNA stability/turnover provides an important mechanism for post-transcriptional control of gene expression [25]. Many studies have reported that frameshift variants can lead to disease by affecting the stability of mRNA [26,27,28]. Therefore, the p.S119Pfs*2 variant may lead to tooth agenesis by affecting the stability of mRNA, but the specific mechanism needs more experiments to verify. Although the results of two missense variants in subcellular localization were consistent with wild-type PAX9, the binding ability to the BMP4 promoter was affected. Subcellular localization and binding to the BMP4 promoter of the p.S119Pfs*2 variant were not consistent with wild-type PAX9. Studies have shown that Pax9 directly activates the Bmp4 promoter, which is very important in tooth development [29,30,31]. Our results suggest that all four variants (p.G64V, p.V117G, p.S119Pfs*2, and p.Y217Lfs*100) affect BMP4 expression, which may be the mechanism of tooth agenesis. However, further functional experiments are needed to clarify its exact mechanism.

An analysis of the missing tooth positions in 151 non-syndromic tooth agenesis patients with *PAX9* variants revealed that the maxillary second molar (84.11%) and mandibular second molar (84.11%) were the most affected tooth positions, followed by the maxillary first molar (74.83%), maxillary second premolar (64.24%), and mandibular central incisor (49.01%). These results suggest that *PAX9* may play an important role in the development of these teeth, especially the second molars. Moreover, we found that the mandibular first premolar (5.96%), mandibular canine (7.62), mandibular lateral incisor (9.60%), and maxillary central incisor (9.93%) were least affected. This finding has not been reported before and indicates that *PAX9* may not be essential during the development of these teeth.

Taken together, we report four novel *PAX9* variants in non-syndromic tooth agenesis, and our results broaden the variant spectrum of *PAX9*. Pattern analysis of non-syndromic tooth agenesis caused by *PAX9* variants can help with clinical diagnosis, treatment, and genetic counseling.

## Figures and Tables

**Table 1:** Functional impact prediction of *PAX9* variants.

Number	Exon	Nucleotide Change	Protein Change	Variation Type	SIFT ^a^	PROVEAN ^b^	PolyPhen-2 ^c^	Fathmm ^d^	Mutation Taster ^e^	ACMG Classification (Evidence of Pathogenicity)
#836 II 2	2	c.350T>G	p. Val117Gly	Missense	0.000 Damaging	−6.789 Deleterious	0.981 (probably damaging)	−5.97 Damaging	Disease-causing	Pathogenic PS2 + PS3 + PM1 + PM2 + PP1 + PP2 + PP3 + PP4
#821 III 1	2	c.352delC	p. Ser119Pro fs*2	Frameshift					Disease-causing	Pathogenic PVS1 + PS3 + PM1 + PM2 + PM4
#622 II 2	2	c.191G>T	p. Gly64Val	Missense	0.000 Damaging	−8.976 Deleterious	1.000 (probably damaging)	−6.84 Damaging	Disease-causing	Pathogenic PS3 + PM1 + PM2 + PP1 + PP2 + PP3
#350 III 1	3	c.648_649insC	p. Tyr217Leu fs*100	Frameshift					Disease-causing	Pathogenic PVS1 + PS3 + PM2 + PM4

^a^ The SIFT score threshold is predefined at 0.05 for binary classification. A protein variant with a score below the threshold is predicted to be damaging; otherwise, it is predicted to be tolerated. ^b^ The PROVEAN score threshold is predefined at −2.5 for binary classification. A protein variant with a score below the threshold is predicted to be deleterious; otherwise, it is predicted to be neutral. ^c^ The PolyPhen-2 analysis appraises a mutation by score as probably damaging (0.909–1), possibly damaging (0.447–0.908), or benign (0–0.446) using the HumVar database. ^d^ Fathmm predicts the functional effects of protein missense variants (damaging vs. tolerated). ^e^ Mutation Taster analysis predicts whether a protein variant is disease-causing (probably deleterious), disease-causing automatic, polymorphism (probably harmless), or polymorphism automatic.

## Data Availability

The data presented in this study are openly available in ClinVar (http://www.ncbi.nlm.nih.gov/clinvar (accessed on 10 February 2022)), Submission name: SUB11740173 and SUB11062545.

## Supplementary Materials

The following supporting information can be downloaded at: https://www.mdpi.com/article/10.3390/ijms23158142/s1.
