# Supplementary material for: Four Novel PAX9 Variants and the PAX9-Related Non-Syndromic Tooth Agenesis Patterns
Source: Int J Mol Sci. 2022 Jul 24;23(15):8142. doi: 10.3390/ijms23158142 (PMC9331840; doi:10.3390/ijms23158142)
Supplement: Supplementary file 1 [file ijms-23-08142-s001.zip › ijms-1830351-supplementary.pdf]

Table S1 Primers used for PCR amplification and PCR conditions

Exon		Primer (5’-3’)	Annealing T (°C)
2	Forward	GGACAGCCCCAGTAGTTAGTA	60°C
	Reverse	AAAGGAAAAAGCCTCAGGTGG	
3	Forward	AAGGTCTAAGCCCTCCAGCT	60°C
	Reverse	CAGTCCACGAAGGATCTG	

Table S2. Summary of human PAX9 variants associated with non-syndromic oligodontia

Exon	Nucleocide change	Protein change	Type of variation		Right quadrants								Left quadrants								Reference
				Max	8	7	6	5	4	3	2	1	1	2	3	4	5	6	7	8	
				Mand	8	7	6	5	4	3	2	1	1	2	3	4	5	6	7	8	
2 (PD)	c.218_219insG	p.Ser74fs	frameshift mutation		*	*	*	*	*							*	*	*	*	*	Stockton (2000) Nat Genet 24, 18
					*	*	*	*					*				*	*	*	*	
					*	*	*	*									*	*	*	*	
					*	*	*	*									*	*	*	*	
					*	*	*	*									*	*	*	*	
					*	*													*	*	
					*	*	*	*									*	*	*	*	
					*	*													*	*	
					*	*	*	*									*	*	*	*	
					*	*	*	*				*	*				*	*	*	*	
					*	*	*	*									*	*	*	*	
					*	*						*	*						*	*	
					*	*	*	*									*	*	*	*	
					*	*	*					*	*					*	*	*	
					*	*	*	*									*	*	*	*	
					*								*							*	
					*	*	*	*									*	*	*	*	
					*	*						*	*						*	*	
					*	*														*	
					*	*						*	*						*	*	
					*	*												*	*	*	
					*	*	*	*											*	*	

2 (PD)	c.340A > T	p.Lys114*	nonsense mutation		*	*	*	*			*			*			*	*	*	*	Nieminen (2001) Eur J Hum Genet 9, 743
					*	*	*											*	*	*	
					*	*					*			*					*	*	
					*	*							*						*	*	
					?	*	*	*						*			*	*	*	?	
					?	*	*										*	*	*	?	
	44-100 kb incl. entire gene		deletion		*	*	*	*	*		*					*	*	*	*	*	Das (2002) Hum Genet 110, 371
					*	*	*	*	*							*	*	*	*	*	
					*	*	*	*	*			*	*			*	*	*	*	*	
					*	*	*	*	*		*	*	*	*		*	*	*	*	*	
4	c.792_793insC	p.Val265fs	frameshift mutation		*	*	*	*	*		*			*			*	*	*	*	Frazier-Bowers (2002) J Dent Res 81, 129
					*	*											*		*	*	
					*	*	*											*	*	*	
					*	*													*	*	
					*	*	*				*						*	*	*	*	
					*	*														*	
					*	*	*	*									*	*	*	*	
					*	*						*	*					*		*	
					*	*	*	*									*	*	*	*	
					*	*													*	*	
					*	*	*											*	*	*	
					*	*													*	*	
					*	*	*	*									*	*	*	*	
					*	*						*	*						*	*	
					*	*	*											*	*	*	
					*	*												*	*	*	
					*	*	*	*									*	*	*	*	
					*	*	*	*									*	*	*	*	
2 (PD)	c.62T > C	p.Leu21Pro	missense mutation		*	*	*	*			*			*		*		*	*	*	Das (2003) Am J Med Genet 118A, 35
					*	*	*	*					*				*	*	*	*	
					*	*	*		*							*		*	*	*	
					*	*						*	*						*	*	
					*		*	*									*	*		*	
					*	*		*				*	*				*		*	*	
					*	*	*	*		*							*	*	*	*	
					*	*	*	*					*					*	*	*	

					*	*	*		*	*						*				*	
					*	*													*	*	
					*						*			*						*	
					*							*	*							*	
					*	*	*	*									*	*	*	*	
					*	*	*					*	*				*	*	*	*	
					*	*	*	*									*	*	*	*	
						*						*	*				*		*		
					*	*	*											*	*	*	
					*	*						*	*						*	*	
2 (PD)	c.175_183delGATACAAins288bp	p.Arg59fs	frameshift mutation		*	*	*	*									*	*	*	*	
					*		*					*						*		*	
					*	*	*	*									*	*	*	*	
					*	*						*	*						*	*	
2 (PD)	c.271A > G	p.Lys91Glu	missense mutation		*	*		*									*		*	*	
					*	*		*			*	*	*				*		*	*	
											*			*							
					*	*														*	
					*															*	
					*	*	*		*	*					*	*			*	*	
					*	*						*	*						*	*	
					*	*	*		*		*			*		*		*	*	*	
					*	*		*				*	*				*		*	*	
					*	*	*	*										*	*	*	
					*															*	
2 (PD)	c.76C > T	p.Arg26Trp	missense mutation		*	*	*			*								*	*	*	Lammi (2003) Eur J Hum Genet 11, 866
					*	*		*											*	*	
					*	*		*		*	*			*	*	*			*	*	
					*	*		*				*	*			*			*	*	
					*	*		*									*	*	*	*	
					*	*		*					*				*		*	*	
					?	*	*	*			*			*			*	*	*	?	
					?	*		*				*					*		*	?	
2	c.151G > A	p.Gly51Ser			*			*			*			*	*		*			*	

(PD)			missense mutation		*			*					*	*			*			*	Mostowska (2003) Eur J Oral Sci 111, 272
2 (PD)	c.83G > C	p.Arg28Pro	missense mutation		*	*	*	*		*					*		*	*	*	*	Jumlongras (2004) Hum Genet 114, 242
					*	*						*	*					*	*	*	
1	c.1A > G	p.Met1Val	missense mutation		*	*	*	*	*							*	*	*	*	*	Klein (2005) J Dent Res 84, 43
					*	*	*	*									*	*	*	*	
					*	*	*	*	*	*					*	*	*	*	*	*	
					*	*	*	*				*	*				*	*	*	*	
2 (PD)	c.108_109insG	p.Ile37fs	frameshift mutation		*	*			*	*		*	*			*	*	*	*	*	Zhao (2005) Zhonghua Kou Qiang Yi Xue Za Zhi 40, 266
					*	*				*		*	*		*				*	*	
2 (PD)	c.139C > T	p.Arg47Trp	missense mutation		*	*	*				*			*		*		*	*	*	
					*	*						*	*						*	*	
2 (PD)	c.259A > T	p.Ile87Phe	missense mutation		*	*	*	*									*	*	*	*	Kapadia (2006) Eur J Hum Genet 14, 403
					*	*	*	*									*	*	*	*	
					*	*	*	*									*	*	*	*	
					*		*	*									*	*	*	*	
2	c.619_621delAT Cins24bp	p.Ile207fs	frameshift mutation		*	*	*	*									*	*	*	*	Mostowska (2006) Eur J Hum Genet 14, 173
					*	*	*	*				*	*				*	*	*	*	
					*	*	*	*									*	*	*	*	
					*	*	*	*				*	*				*	*	*	*	
					*	*	*	*									*	*	*	*	
					*	*	*	*				*	*				*	*	*	*	
					*	*	*	*									*	*	*	*	
					*	*	*	*				*	*				*	*	*	*	
					*	*	*	*									*	*	*	*	
					*	*	*	*				*	*				*	*	*	*	
2	c.433C > T	p.Gln145*	nonsense mutation		*	*	*								*	*	*	*	*	*	Hansen (2007) Eur J Oral Sci 115, 330
					*	*		*			*	*	*	*			*		*	*	
					*															*	
					*	*													*	*	
					*			*									*			*	
					*			*									*			*	
					*	*		*									*	*	*	*	
					*	*		*									*		*	*	

					*	*	*	*									*		*	*	
					*	*							*						*	*	
3	c.718G > C	p.Ala240Pro	missense mutation							*	*			*	*						Wang (2009) Hua Xi Kou Qiang Yi Xue Za Zhi 27, 606
										*	*	*	*	*	*						
2 (PD)	c.16G > A	p.Gly6Arg	missense mutation		*												*			*	Wang (2009) Cells Tissues Organs 189, 80
					*							*	*							*	
	c.128G > A and c.129C > A	p.Ser43Lys			*	*	*		*							*		*	*	*	
					*	*	*												*	*	
					*					*					*					*	
					*	*													*	*	
5'-UTR	g.-1258G > A				*	*	*											*	*	*	Mendoza-Fandino (2011) Clin Genet 80, 265
					*	*	*						*					*	*	*	
					*		*											*		*	
					*		*											*		*	
					*	*	*											*	*	*	
					*	*	*						*					*	*	*	
					*															*	
					*	*													*	*	
					*		*	*		*	*			*	*				*	*	
					*	*				*				*					*	*	
					*	*	*	*			*			*		*		*	*	*	
					*	*		*				*	*				*		*	*	
					*	*											*	*			
					*	*													*	*	
					*	*	*		*							*		*	*	*	
					*	*		*				*					*		*	*	
					*	*	*		*							*		*	*	*	
					*	*		*				*	*				*		*	*	
2 (PD)	c.321_322insG	p.Ala108fs	frameshift mutation		*	*	*	*									*	*	*	*	Suda (2011) J Dent Res 90, 382
					*	*	*	*									*	*	*	*	
					*	*	*											*	*	*	
					*	*	*	*										*	*	*	
					*	*	*	*									*	*	*	*	
					*	*	*	*									*	*	*	*	
					*	*	*	*									*	*	*	*	

					*	*	*						*					*	*	*	
2 (PD)	c.80T > C	p.Leu27Pro	missense mutation		*	*	*			*					*			*	*	*	Liang (2012) Arch Oral Biol 57, 784
					*	*	*											*	*	*	
					*	*	*			*							*	*	*	*	
					*	*	*					*									
2 (PD)	c.86T > C	p.Ile29Thr	missense mutation		*	*	*	*		*					*			*	*	*	
					*	*				*					*				*	*	
					*	*	*			*				*	*			*	*	*	
					*	*													*	*	
2 (PD)	c.43T > A	p.Phe15Ile	missense mutation		*			*									*			*	Wang (2012) PLoS One 7, e51533
					*							*	*							*	
					?	*		*								*	*		*	?	
					?	*		*				*	*						*	?	
2 (PD)	c.218_219insG	p.Ser74fs	frameshift mutation		?	*	*	*	*		*			*		*	*	*	*	?	Zhu (2012) Mutagenesis 27, 313
					?	*	*			*								*	*	?	
2	c.480C > G	p.Tyr160*	nonsense mutation		?	*	*	*	*	*					*	*	*	*	*	?	
					?	*	*	*			*	*	*	*			*	*	*	?	
2 (PD)	c.140G > C	p.Arg47Pro	missense mutation		*	*											*		*	*	Arte (2013) PLoS One 8, e73705
					*			*				*	*				*			*	
2 (PD)	c.167T > C	p.Ile56Thr	missense mutation		*	*	*											*	*	*	
					*														*	*	
2 (PD)	c.340A > T	p.Lys114*	nonsense mutation		*	*	*											*	*	*	
					*														*	*	
2	c.406C > T	p.Gln136*	nonsense mutation		*	*	*	*										*	*	*	
					*	*													*	*	
2	c.503C > G	p.Ala168Gly	missense mutation		*															*	Boeira Junior (2013) J Oral Pathol Med 42, 99
					*			*									*			*	
					*															*	
					*			*			*						*			*	
								*	*							*	*				
								*	*							*	*				
					*			*									*			*	
																	*				
								*									*				
																				*	
					*			*	*							*	*			*	

								*	*								*				
					*			*									*				
								*													
								*									*		*	*	
2 (PD)	c.353_354insTGCC	p.Ser119fs	frameshift mutation		?		*	*									*	*	*	?	Mostowska (2013) Clin Genet 84, 429
					?	*						*	*						*	?	
2 (PD)	c.73-75delATC	p.Ile25del	frameshift mutation		*	*	*											*	*	*	Mitsui (2014) J Dent Res 93, 245
					*	*													*	*	
2 (PD)	c.146C > T	p.Ser49Leu	missense mutation		*	*	*	*									*	*	*	*	
					*	*						*	*						*	*	
IVS2	g.9527G > T		splicing		*	*	*	*			*			*			*	*	*	*	Šerý (2015) Eur J Oral Sci 123, 65
					*	*						*							*	*	
					*	*	*	*			*			*			*	*	*	*	
					*	*													*	*	
					*	*	*	*									*	*	*	*	
					*	*		*									*		*	*	
2 (PD)	c.59C > T	p.Pro20Leu	missense mutation		*	*		*		*	*			*	*		*		*	*	Thimmegowda (2015) J Clin Diagn Res 9, ZD08
					*	*		*		*					*		*		*	*	
					*						*			*						*	
					*															*	
					*					*	*			*						*	
										*	*	*	*	*	*		*			*	
					*						*			*						*	
					*															*	
2	c.592_596dup	p.Asp200fs	frameshift mutation		*	*	*		*							*		*	*	*	Haddaji Mastouri (2016) Arch Oral Biol 71, 110
					*	*		*				*	*						*	*	
						*										*			*	*	
					*	*						*	*						*	*	
					*	*	*	*	*								*	*	*	*	
					*							*	*							*	
					*															*	
					*	*													*	*	
1	c.2T > G	p.Met1Arg	missense mutation		*	*	*			*	*	*	*	*	*				*	*	Liang (2016) Arch Oral Biol 61, 144
					*	*	*					*	*					*	*	*	
					*	*	*	*									*	*	*	*	

					*	*	*											*	*	*	
					*	*	*	*							*			*	*	*	
					*	*	*											*	*	*	
					*	*	*	*				*	*				*		*	*	
					*	*		*	*		*	*		*				*	*	*	
					*	*	*	*										*	*	*	
					*	*						*	*						*	*	
2 (PD)	g.10672A > T	p.Asn116Ile	missense mutation								*										Shahid (2016) Eur J Med Genet 59, 377
								*													
2 (PD)	c.289_296del	p.Ile97fs	frameshift mutation		Cannot find more details																Yu (2016) Am J Hum Genet 99, 195
1	c.3G > A	p.Met1Ile	missense mutation		*	*	*	*	*	*	*	*	*	*			*	*	*	*	Sarkar (2017) Gene 635, 69
					*	*	*					*	*				*	*	*	*	
					*	*		*							*		*	*	*	*	
					*	*	*											*	*	*	
					*	*	*	*				*	*			?		*	*	*	
					*		*											*	*	*	
					*	*				*					*		*	*	*	*	
					*	*						*							*	*	
					*	*	*	*							*			*	*	*	
					*	*	*	*									*	*	*	*	
					*	*		*									*		*	*	
					*	*						*	*					*	*		
2 (PD)	c.76C > T	p.Arg26Trp	missense mutation			*	*							*			*	*	*		Wong (2018) J Dent Res 97, 155
						*						*	*			*			*		
2 (PD)	c.140G > C	c.140G>C	missense mutation			*	*	*	*							*	*	*	*		
						*	*	*	*			*	*	*		*	*	*	*		
2 (PD)	c.146delC	p.Ser49fs	frameshift mutation			*	*	*		*	*						*	*	*		
						*	*			*	*	*						*	*		
2 (PD)	c.167T > C	p.Ile56Asn	missense mutation			*	*	*	*							*	*	*	*		
						*		*									*		*		
2 (PD)	c.185_189dup	p.Gly64fs	frameshift mutation			*	*				*			*			*	*	*		
						*	*				*			*					*		
2	c.194C > A	p.Ser65*				*	*	*	*	*				*	*	*	*	*	*		

(PD)			nonsense mutation			*	*	*				*	*				*	*	*		
2 (PD)	c.218dupG	p.Ser74fs	frameshift mutation			*	*	*	*		*			*		*	*	*	*		
						*		*		*					*		*		*		
2 (PD)	c.256_262dup	p.Arg88fs	frameshift mutation			*	*	*									*	*	*		
						*	*	*											*		
2 (PD)	c.271A > T	p.Lys91*	c			*	*	*		*		*	*	*			*	*	*		
						*				*	*	*	*	*	*				*		
2 (PD)	c.322G > C	p.Ala108Pro	missense mutation				*										*	*	*		
						*						*	*						*		
2	c.592delG	p.Val198fs	frameshift mutation		Cannot find more details																
1	c.2T > A	p.Met1*	nonsense mutation		*	*	*	*	*							*	*	*	*	*	Koskinen (2019) Clin Oral Investig 23, 4107
					*	*	*	*	*							*	*	*	*	*	
						*	*	*		*	*						*	*	*		
						*						*	*						*		
						*	*	*	*		*			*			*	*	*		
						*	*	*				*	*				*	*	*		
2(PD) 3	c.331G > A and c.718G > C	p.Val111Met p.Ala240Pro	missense mutation			*	*		*		*					*					Zhang(2019) Oral Dis 25, 234
2 (PD)	c.211_212insA	p.Ile71fs	frameshift mutation			*	*		*	*		*	*	*	*	*		*	*		Sun(2021) Oral Dis 27,1468
						*	*					*	*				*	*	*		
2 (PD)	c.229C > G	p.Arg77Gly	missense mutation			*			*		*			*	*	*			*		
						*	*	*									*	*	*		
2 (PD)	c.236_237insAC	p.Ter80fs	frameshift mutation			*	*	*	*						*		*	*	*		
						*	*	*				*	*				*	*	*		
2 (PD)	c.59C > T	p.Pro20Leu	missense mutation		*	*		*			*	*	*				*		*	*	Intarak (2022) Eur J Oral Sci 130,2
					*	*							*						*	*	
2 (PD)	c.230G>A	p.Arg77Gln	missense mutation		*	*	*											*	*	*	
					*	*	*											*	*	*	
					*	*	*											*	*	*	
					*	*	*											*	*	*	
2	c.491-510delGCCCTATCACGGCGGCGGCC	p.Pro165fs	frameshift mutation			*	*	*	*		*	*	*	*		*	*	*	*		Sun(2022) Oral Dis May 20
						*	*	*				*	*				*	*	*		
						*	*	*				*				*	*	*	*		
						*	*	*				*	*				*	*	*		

						*	*	*	*	*		*	*		*	*	*	*	*		
						*	*	*			*	*	*	*		*	*	*	*		
						*	*	*			*	*	*	*	*	*	*	*	*		
						*	*	*	*		*	*	*	*			*	*	*		
						*	*	*	*	*	*	*	*	*	*	*	*	*	*		
						*	*	*	*	*	*	*	*	*	*	*	*	*	*		
2 (PD)	c.350T＞G	p.Val117Gly	missense mutation																		this study
						*													*		
						*		*											*		
						*													*		
3	c.648_649insC	p.Tyr217Leufs*100	frameshift mutation			*	*	*	*		*			*	*		*	*	*		
						*	*	*				*	*				*	*	*		
2 (PD)	c.352delC	p.Ser119Profs*2	frameshift mutation			*	*	*	*	*	*	*	*	*	*	*	*	*	*		
						*	*	*		*		*	*		*		*	*	*		
2 (PD)	c.191G＞T	p. Gly64Val	missense mutation			*	*	*		*	*		*	*	*		*	*	*		
						*	*	*		*		*	*	*	*		*	*	*		
